# Effects of Structured Physical Therapy on Spinal Alignment in Idiopathic Scoliosis: A 12-Month Prospective Study

**DOI:** 10.3390/diagnostics15212747

**Published:** 2025-10-30

**Authors:** Brigitte Osser, Csongor Toth, Carmen Delia Nistor-Cseppento, Iosif Ilia, Gyongyi Osser, Mariana Cevei, Cristina Aur, Roland Fazakas, Laura Ioana Bondar

**Affiliations:** 1 Doctoral School of Biomedical Sciences, University of Oradea, 410087 Oradea, Romania; brigitte.osser@uav.ro (B.O.); dcseppento@uoradea.ro (C.D.N.-C.); bondar.lauraioana@student.uoradea.ro (L.I.B.); 2 Faculty of Physical Education and Sport, “Aurel Vlaicu” University of Arad, 310130 Arad, Romania; iosif.ilia@uav.ro (I.I.); gyongyi.osser@uav.ro (G.O.); 3 Department of Psycho Neuroscience and Recovery, Faculty of Medicine and Pharmacy, University of Oradea, 410087 Oradea, Romania; cevei_mariana@uoradea.ro; 4 Department of Surgical Disciplines, Faculty of Medicine and Pharmacy, University of Oradea, 410073 Oradea, Romania; d.aur@uoradea.ro; 5 Department of Biology and Life Sciences, Faculty of Medicine, “Vasile Goldiș” Western University of Arad, 310025 Arad, Romania; fazakas.roland@uvvg.ro; 6 Doctoral School of Medicine, “Vasile Goldiș” Western University of Arad, 310025 Arad, Romania

**Keywords:** Cobb angle, exercise therapy, physical therapy modalities, posture, scoliosis, spinal curvatures, treatment outcome

## Abstract

**Background:** Scoliosis, a three-dimensional spinal deformity, can impair posture, function, and quality of life. Conservative approaches such as structured physical therapy are widely used, but evidence from long-term, real-world settings remains limited. In this study, “structured” refers to a standardized, supervised program delivered three times per week, incorporating postural correction, Klapp-based positioning, core strengthening, flexibility training, and progressive intensity adjustments rather than ad hoc or unsupervised exercise. **Methods:** In this 12-month longitudinal study, 240 patients aged 15–19 years with idiopathic scoliosis (Cobb angle 15–25°) were enrolled; 222 completed the program and were included in analyses. All participants were near or beyond skeletal maturity, representing a population with low residual risk of curve progression. Spinal alignment was assessed by Cobb angle at baseline, 6 months, and 12 months, with monthly clinical evaluations to track progress. Outcomes were analyzed by baseline severity, age, sex, and skeletal maturity (Risser stage) using paired-samples t-tests, repeated-measures ANOVA, subgroup analyses, and multivariable regression. Clinically meaningful improvement was defined as a ≥20% reduction in Cobb angle from baseline. **Results:** Mean Cobb angle decreased from 18.59° at baseline to 14.85° at 12 months (Δ = −3.74°, 20.3% relative reduction; *p* < 0.001). The largest improvement occurred in the first 6 months (−3.04°, *p* < 0.001), followed by a smaller gain between months 6 and 12 (−0.70°, *p* = 0.012). Patients with milder baseline curves improved more than those with greater deviations (4.45° vs. 3.21°, *p* < 0.001). Monthly clinical follow-ups suggested gradual improvement between radiographic assessments, though detailed statistical analyses were limited to baseline, 6 months, and 12 months. In multivariable regression, baseline severity remained the only significant predictor of improvement (*p* < 0.001), while age, sex, and Risser stage were not significant (all *p* > 0.05). Overall, 24.3% of participants achieved clinically meaningful improvement. **Conclusions:** A structured 12-month physical therapy program produced significant, sustained improvements in spinal alignment across diverse patient groups. Early intervention in milder curves maximized benefit. These findings support physiotherapy exercise programs as an effective conservative option for scoliosis management.

## 1. Introduction

Scoliosis is a three-dimensional spinal deformity defined by a lateral curvature of ≥10° measured with the Cobb method on standing radiographs; it is relatively common in adolescents (≈2–3% prevalence) and may progress during periods of rapid growth and, in some cases, into adulthood [1,2,3]. While its etiology is often multifactorial—encompassing idiopathic, congenital, and neuromuscular causes—its progression can lead to postural imbalance, reduced respiratory function, chronic pain, and diminished quality of life if left untreated [4,5,6].

Management strategies for scoliosis vary according to curve magnitude and risk of progression, ranging from observation in mild curves, to bracing in progressive moderate curves, and surgical correction in severe cases [7]. Increasingly, conservative interventions—particularly physiotherapeutic scoliosis-specific exercise (PSSE) programs such as the Schroth, SEAS (Scientific exercise approach to scoliosis), or Lyon methods—are emphasized in international guidelines for their potential to improve spinal alignment, muscular balance, and functional outcomes without the risks associated with surgery [8,9,10,11,12]. The term PSSE refers broadly to an umbrella category of physiotherapy approaches that share common corrective principles but differ in methodology and school-specific protocols (e.g., Schroth, SEAS, Lyon). In this context, “structured” denotes a standardized, supervised program with defined frequency, duration, exercise components (e.g., postural correction, core strengthening, flexibility training, and scoliosis-specific corrective exercises), and progressive intensity adjustments, distinguishing it from ad hoc or unsupervised exercise.

However, the structured physiotherapy program applied in the present study was not derived from any single PSSE or Schroth-based method. Instead, it integrated postural correction, Klapp-based positioning, core stabilization, flexibility training, and controlled breathing techniques commonly used in conservative physiotherapy practice. This clarification ensures conceptual accuracy and distinguishes our intervention from curve-pattern–specific PSSE approaches.

A growing body of evidence suggests that the rate and magnitude of improvement following conservative treatment may be influenced by several factors. Temporal dynamics, such as the early rapid gains observed during initial months of therapy followed by a plateau, have been linked to neuromuscular adaptation and structural consolidation [8,13]. Baseline curve severity may also predict treatment outcomes, with larger curves showing greater potential for absolute correction due to a larger margin for change [14,15,16]. Additionally, demographic factors—including age and sex—may influence flexibility, compliance, and physiological responsiveness, though findings remain inconsistent across studies [17,18,19].

Sustained improvement is a key goal in scoliosis rehabilitation, as maintaining correction over time is essential for long-term management. Beyond statistical significance, a ≥20% reduction in Cobb angle is commonly accepted as a clinically meaningful benchmark for evaluating treatment effectiveness [14,20,21,22]. Importantly, a recent comprehensive meta-analysis confirmed that exercise-based corrective therapy can produce clinically meaningful improvements in adolescent idiopathic scoliosis (AIS), consolidating the role of therapeutic exercise as a cornerstone of conservative management [23].

Despite the growing body of literature on exercise-based scoliosis management, there remain notable gaps in understanding the month-to-month progression of spinal alignment, the durability of changes beyond initial improvements, and the extent to which baseline severity or demographic factors influence long-term outcomes. Furthermore, most existing studies have been conducted in highly controlled research settings or specialized rehabilitation centers, limiting generalizability to routine clinical practice. There is a need for longitudinal data from real-world physical therapy settings, where adherence, patient diversity, and therapist variability more closely reflect actual clinical environments.

The present study addresses these gaps by evaluating a 12-month structured physical therapy program in patients with scoliosis in real-world clinical practice. The central hypothesis is that this program leads to significant and sustained improvement in spinal alignment, with the greatest gains occurring during the first six months, independent of age, sex, or skeletal maturity.

## 2. Materials and Methods

### 2.1. Study Design and Setting

This was a prospective, longitudinal observational cohort study without a control group, designed to evaluate the effects of a structured physical therapy program on spinal alignment in patients with scoliosis over a 12-month period. Radiographic assessments were performed at baseline, 6 months, and 12 months to measure spinal alignment, while monthly clinical evaluations—including scoliometer measurements, postural observation, trunk mobility testing, and adherence checks—were conducted to track interim progress and ensure adherence. This approach allowed for both detailed temporal monitoring and minimization of radiation exposure.

A concurrent control group was not included for ethical and practical reasons: all eligible patients were referred for conservative therapy, and withholding treatment or assigning patients to a “no-intervention” arm was not feasible in this real-world clinical setting. The study therefore prioritized a pragmatic design, focusing on within-patient change over time.

The research was conducted across multiple specialized rehabilitation facilities with established expertise in conservative scoliosis management. Participating centers included Ilimed Kinetica, Timișoara; Davima Clinic, Arad; and Dr. Tuduce Medical Rehabilitation Clinic, Arad. Each center provides individualized, supervised physiotherapeutic interventions delivered by licensed physical therapists formally trained in scoliosis rehabilitation. The therapists had professional experience with principles derived from several recognized PSSE schools—such as Schroth and SEAS—but the structured program applied in this study was not based on any single school-specific protocol. Instead, it integrated postural correction, Klapp-based positioning, core stabilization, flexibility training, and controlled breathing techniques consistent with evidence-based conservative physiotherapy practice. All radiographs were obtained in dedicated clinical evaluation units equipped with standardized imaging facilities, ensuring uniform measurement conditions across the study period.

### 2.2. Study Population

A total of 240 patients diagnosed with idiopathic scoliosis were consecutively enrolled between August 2023 and August 2025 across three specialized rehabilitation clinics with established expertise in scoliosis management. Recruitment over this two-year period resulted in approximately 80 patients per center (≈40 per center per year), reflecting the routine clinical volume of these facilities.

Eligibility criteria were:

- Radiographically confirmed scoliosis with a Cobb angle between 15° and 25° at baseline assessment.
- Age ≥ 15 years (this threshold was deliberately chosen to ensure sufficient skeletal maturity and consistency in outcome evaluation. While literature indicates that conservative interventions can be beneficial from approximately 10 years of age, especially during growth spurts, we focused on older adolescents and young adults to minimize variability linked to skeletal immaturity and to strengthen the reliability of longitudinal comparisons.
- Ability to attend regular therapy sessions for 12 months.
- No prior spinal surgery.

Exclusion criteria included:

- Neuromuscular or congenital scoliosis (excluded due to differing etiology, structural characteristics, and progression patterns, as well as differential response to conservative therapy).
- Severe cardiorespiratory or musculoskeletal conditions limiting participation.
- Concurrent participation in another scoliosis-specific intervention.
- Incomplete follow-up data.

Patients older than 18 years were also eligible, as conservative therapy is frequently applied in late adolescents and adults with scoliosis, either to improve postural alignment and trunk symmetry or to address pain and quality-of-life concerns that persist beyond skeletal maturity.

### 2.3. Intervention Protocol

The structured physical therapy program lasted 12 months and emphasized spinal alignment, postural control, and neuromuscular re-education. In this context, “structured” refers to a standardized, supervised program with predefined frequency (three sessions per week), duration (45–60 min), and exercise components (warm-up, postural correction, strengthening, flexibility, corrective exercises, breathing, and cool-down), combined with progressive intensity adjustments and a mandatory home-exercise component.

**Figure 1:**
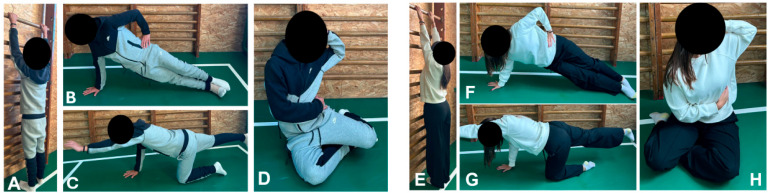
Representative exercises included in the structured physical therapy protocol. Panels (**A**–**D**) show a male participant; panels (**E**–**H**) show a female participant. Exercises illustrated: (**A**,**E**) Wall-supported spinal elongation; (**B**,**F**) Side plank; (**C**,**G**) Bird-dog stabilization; (**D**,**H**) corrective posture emphasizing elongation, derotation, and controlled breathing. These exercises are commonly used in physiotherapy practice for scoliosis management but are not derived from any single PSSE or Schroth method.

Patients attended supervised sessions three times per week, each lasting approximately 45–60 min, delivered by licensed physiotherapists trained in scoliosis-specific management. The intervention was not derived from any single Schroth or PSSE school. Instead, it integrated general corrective exercise principles drawn from Klapp-based positioning, core stabilization, flexibility training, and controlled breathing techniques commonly used in conservative physiotherapy for scoliosis. Although the exercises incorporated elements such as elongation, derotation, and postural correction that are conceptually similar to those used in PSSE approaches, the program was designed as a pragmatic physiotherapy protocol rather than a curve-pattern–specific or school-based PSSE method. Each session followed a standardized structure:

- Warm-up (5–10 min): light aerobic activity and mobility drills to prepare the spine and surrounding musculature.
- Postural correction and elongation (10–15 min): wall-supported spinal elongation, mirror feedback exercises, and corrective positioning tailored to curve type (Figure 1A,E).
- Core and paraspinal strengthening (15–20 min): isometric trunk extensions, side planks (Figure 1B,F), bridging with leg lifts, bird-dog exercises (Figure 1C,G), and resisted band work to improve trunk stability.
- Flexibility training (10 min): static and dynamic stretching of hamstrings, hip flexors, and thoracic spine, with emphasis on asymmetrical stretches when indicated.
- Scoliosis-specific corrective exercises (10–15 min): corrective postures such as side-shifting, derotation with rotational breathing (Figure 1D,H), and three-dimensional elongation.
- Breathing and proprioceptive drills (5 min): segmental breathing with corrective alignment, balance exercises on unstable surfaces, and trunk stabilization tasks.
- Cool-down (5 min): relaxation and gentle stretching to consolidate postural correction.

Exercises were individualized according to each patient’s predominant curve pattern (thoracic, thoracolumbar, lumbar, or double major), with corrective movements adapted to the curve’s convexity and region of maximal deviation. This ensured that the “curve-type–specific” component of the program corresponded to each participant’s baseline spinal morphology.

Exercise intensity and complexity were progressively increased every 4–6 weeks by adjusting repetitions, duration of holds, resistance, and transitioning from static to dynamic tasks. A daily home program of 15–20 min was prescribed, focusing on corrective breathing, posture-holding, and stretching. The home program was considered an essential component, and patients were provided with written and illustrated instructions to ensure correct execution [24,25]. Adherence was systematically monitored through therapist-maintained attendance logs. Missed sessions were documented, and patients were encouraged to complete make-up sessions either in clinic or at home with guided instructions. Overall adherence exceeded 90% among completers.

### 2.4. Data Collection Instruments

Data collection was performed using standardized clinical and radiographic assessment tools to ensure measurement reliability and comparability over time. The primary outcome measure was the Cobb angle, determined from standing posteroanterior full-spine radiographs [26,27]. Radiographs were obtained at baseline, 6 months, and 12 months using a standardized positioning protocol to minimize variability in spinal curvature measurement. Cobb angle determination was performed by trained musculoskeletal radiologists using a digital measurement system (RadiAnt DICOM Viewer, version 2023.1; Medixant, Poznań, Poland), which allows precision to 0.1°. Inter-rater reliability was confirmed prior to study initiation, with an intraclass correlation coefficient (ICC) exceeding 0.95.

Skeletal maturity was also evaluated using the Risser score, determined from pelvic radiographs obtained at baseline. Risser staging (0–5) was based on the degree of iliac apophysis ossification and served as an objective indicator of bone maturation [28,29].

Trunk rotation was assessed with a handheld scoliometer during the Adams forward bend test. Examinations were performed by trained physiotherapists following a standardized protocol: patients stood with feet shoulder-width apart, knees extended, and arms relaxed and flexed forward until the back was parallel to the floor. The scoliometer was placed perpendicular to the spine at the level of maximal rib hump (thoracic) or lumbar prominence, and three consecutive readings were averaged. When both thoracic and lumbar prominences were present, the maximal angle of trunk rotation (ATR) was recorded as the primary measure. Assessments were conducted at baseline, 6 months, and 12 months [30,31].

Finally, a structured case record form was used to collect demographic data (age, sex, residential background), clinical characteristics (baseline Cobb angle, scoliosis type), and therapy adherence (session attendance logs). Monthly follow-up visits included non-invasive clinical evaluations—such as scoliometer checks, postural observation, trunk mobility assessment, and review of adherence logs—together with documentation of therapy participation. These measures allowed continuous monitoring of postural changes and treatment adherence between radiographic assessments.

At monthly follow-up visits, postural alignment was systematically assessed using a standardized non-radiographic protocol [32,33]. Patients stood barefoot in a neutral position (feet shoulder-width apart, knees extended, arms relaxed) against a posture grid to allow serial comparison. Trained physiotherapists evaluated shoulder level, pelvic obliquity, coronal trunk shift (plumb line from C7 relative to gluteal cleft), waist symmetry, and scapular/rib cage prominence by visual inspection and calibrated photography. In addition, sagittal alignment was appraised (forward head, thoracic kyphosis, lumbar lordosis), and trunk mobility was assessed through lateral flexion and rotation tests. ATR was measured monthly with a scoliometer during the Adams forward-bend test, following the same procedure as baseline. To ensure consistency, all assessments were performed in the same environment, with floor markers and fixed camera distance when photographs were taken. Meaningful month-to-month changes were predefined as ≥2° for ATR, ≥5 mm for shoulder or pelvic asymmetry, or ≥5 mm trunk shift; smaller differences were considered within normal variability. These standardized evaluations provided continuous monitoring of postural change between radiographic assessments.

It is important to note that monthly evaluations were clinical and descriptive, intended to monitor progress and detect potential regressions, whereas formal statistical analyses were applied only to radiographic outcomes at baseline, 6 months, and 12 months.

### 2.5. Ethical Considerations

Ethical approval for this study was obtained from the Research Ethics Committee of the Faculty of Medicine and Pharmacy, University of Oradea (Approval No. CEFMF/1, 27 June 2024). In addition, site-level administrative approvals were granted by each participating rehabilitation clinic: Ilimed Kinetica, Timișoara (Approval No. 1/12 June 2024), Davima Clinic, Arad (Approval No. 19/26 February 2024), and Dr. Tuduce Medical Rehabilitation Clinic, Arad (Approval No. 12/3 October 2023). We clarify that in Romania, private clinics do not have independent ethics committees; therefore, the University of Oradea approval served as the formal ethical authorization for the entire study, while the clinics’ approvals confirmed local compliance and permission to conduct research activities on site. All procedures adhered to the principles of the Declaration of Helsinki and national regulations on biomedical research involving human participants.

Written informed consent was obtained from all participants; for minors, parental or legal guardian consent was secured in addition to the participant’s own assent. Confidentiality and anonymity were strictly maintained, with all data stored in password-protected files accessible only to the research team. Participants were explicitly informed of their right to withdraw at any time without penalty or impact on their ongoing medical care.

To minimize radiation exposure, radiographic assessments were restricted to three standardized time points (baseline, 6 months, and 12 months), while monthly interim evaluations relied exclusively on non-invasive clinical assessments.

### 2.6. Data Analysis

All statistical analyses were performed using IBM SPSS Statistics, version 29.0 (IBM Corp., Armonk, NY, USA). Descriptive statistics, including means, standard deviations, frequencies, and percentages, were calculated to summarize baseline characteristics and outcome measures. Because this was an observational cohort without a control group, the primary focus was on within-patient change across the 12-month period.

Overall treatment effects were evaluated using paired-samples t-tests to compare pre–post changes in Cobb angle. Temporal patterns of improvement were examined using repeated-measures ANOVA across baseline, 6 months, and 12 months, with Bonferroni-adjusted post hoc tests applied where appropriate. Differences in improvement by baseline severity were analyzed using independent-samples t-tests, while the influence of age and sex was assessed with a two-way ANOVA. In addition, skeletal maturity was evaluated using the Risser score, and group comparisons were conducted with one-way ANOVA to determine whether outcomes varied by bone maturation stage.

To account for potential confounding, a multiple linear regression was performed with change in Cobb angle (baseline → 12 months) as the dependent variable and baseline severity, age, sex, and Risser stage as predictors. Given the numerical imbalance between skeletal maturity groups, subgroup analyses were also conducted. Patients were stratified by Risser stage into two groups: high growth potential (Risser 0–1) and near skeletal maturity (Risser 4–5). Between-group differences in Cobb angle change over 12 months were evaluated using linear models adjusted for baseline Cobb angle, age, and sex.

Month-to-month consistency of improvement was evaluated descriptively using clinical follow-up data, with radiographic assessments at 6 and 12 months providing validation of trends. Clinically meaningful improvement was defined as a reduction of at least 20% in Cobb angle from baseline, and proportions were calculated accordingly. Effect sizes were reported as Cohen’s d, Cohen’s dz, standardized response mean, or partial η^2^, depending on the analysis performed.

Normality of distributions was tested using the Shapiro–Wilk test. For variables that did not meet normality assumptions, non-parametric alternatives, including the Wilcoxon signed-rank test and Mann–Whitney U test, were applied as sensitivity analyses. To strengthen robustness, bootstrapped 95% confidence intervals (1000 resamples) were additionally calculated for primary outcomes. Statistical significance was set at *p* < 0.05, with Bonferroni correction applied for multiple comparisons.

No a priori sample size calculation was performed because the study was designed pragmatically to include all consecutive eligible patients over a two-year period across the three participating clinics. Post hoc, based on the observed standard deviation of paired Cobb angle change scores (≈0.90°), we estimated that with α = 0.05 (two-sided) and 80% power, the minimum required sample size to detect mean pre–post changes of 0.5°, 1.0°, and 2.0° would be approximately 26, 7, and 2 patients, respectively. Given that 222 patients completed the study, this cohort was more than adequately powered to detect clinically meaningful differences.

### 2.7. Hypotheses of the Study

In line with the central hypothesis introduced earlier—that a structured 12-month physical therapy program would result in significant and sustained improvement in spinal alignment—several specific hypotheses were formulated. It was expected that:

1. Spinal deviation would decrease significantly from baseline to Month 12;
2. The greatest rate of improvement would occur within the first six months, corresponding to early neuromuscular adaptation;
3. Patients with greater baseline deviation would show larger absolute changes;
4. Improvements would remain stable across consecutive months without regression;
5. Outcomes would be independent of age and sex, demonstrating broad applicability.

These hypotheses collectively guided the statistical analyses and interpretation of results.

## 3. Results

**Figure 2:**
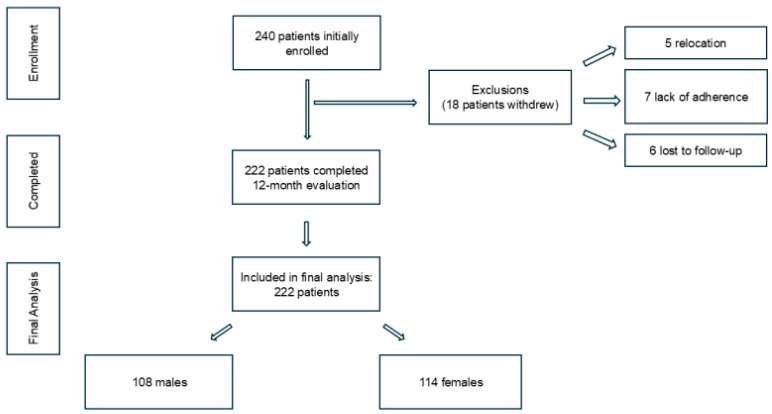
Flow diagram of patient enrollment, follow-up, and inclusion in the final analysis.

A total of 240 patients were enrolled, of whom 18 withdrew (5 relocated, 7 due to non-adherence, 6 lost to follow-up). The remaining 222 participants (92.5%) completed the 12-month program and were included in the analysis (Figure 2).

For subgroup analyses, patients were categorized by age (<18 vs. ≥18 years) and skeletal maturity (Risser stage). Baseline demographic and clinical characteristics are summarized in Table 1. Curve pattern distribution at baseline was as follows: 45.9% thoracic, 31.1% thoracolumbar, 17.1% lumbar, and 5.9% double major curves. As expected, the cohort included more patients with advanced skeletal maturity (Risser 4–5, 41.4%) compared with those with high growth potential (Risser 0–1, 18.9%). This imbalance was considered in subsequent subgroup and interaction analyses.

### 3.1. Overall Improvement in Spinal Alignment

Cobb angle decreased significantly over the 12-month program (Table 2), with mean values falling from 18.59° at baseline to 14.85° at Month 12 (−3.74°, 20.3% reduction, *p* < 0.001). Most of the improvement occurred in the first 6 months (−3.04°), followed by a smaller but sustained decrease in the second half (−0.70°).

Although the average absolute reduction (3.74°) did not reach the ≥5° minimum clinically important difference proposed in some guidelines, it clearly exceeded the 2° benchmark considered relevant in non-surgical management. Furthermore, nearly one-quarter of patients (24.3%) achieved a ≥20% reduction in Cobb angle, a threshold widely recognized in scoliosis rehabilitation research as clinically meaningful, particularly in older adolescents and young adults with limited remaining growth potential.

**Figure 3:**
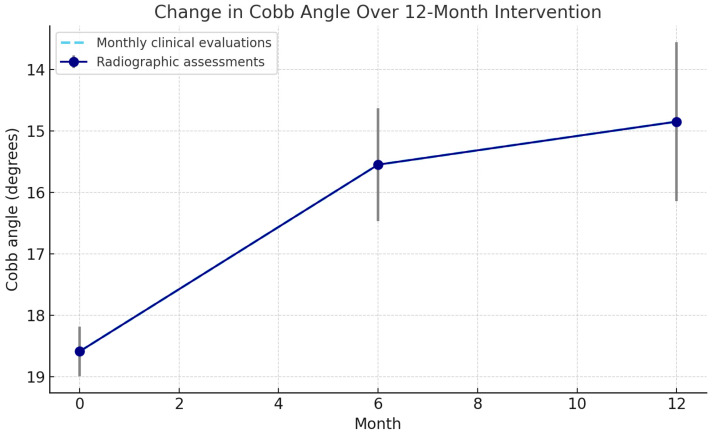
Change in mean Cobb angle across the 12-month intervention. Solid points with confidence intervals represent radiographic assessments at baseline, 6 months, and 12 months. The dashed line indicates monthly clinical evaluations. Note: The y-axis is oriented so that lower Cobb-angle values (indicating improvement) appear higher on the plot; thus, the upward trend represents curve correction rather than progression.

Normality assumptions were not met, but non-parametric analyses confirmed consistent results (*p* < 0.001). Monthly clinical evaluations showed steady improvements, with the steepest reduction in the first 6 months and a plateau thereafter (Figure 3).

### 3.2. Temporal Pattern of Improvement

Cobb angle reduction was greatest during the first 6 months (−3.0°; 16.3%) and slowed thereafter (−0.7° between Months 6–12), indicating a plateau (Figure 3). Repeated-measures ANOVA confirmed a significant time effect (*p* < 0.001), with Bonferroni-adjusted comparisons showing significant reductions from baseline to 6 and 12 months, and a smaller but significant change between 6 and 12 months (Table 3). Monthly clinical evaluations followed the same trajectory, with no evidence of regression.

### 3.3. Improvement by Baseline Severity

When stratified by baseline Cobb angle, patients with lower initial deviation (<18.6°) showed greater improvement (4.45°) than those with higher deviation (≥18.6°, 3.21°). This difference was statistically significant (*p* < 0.001) with a large effect size (Table 4).

### 3.4. Subgroup and Interaction Analyses by Skeletal Maturity

Patients were stratified by Risser stage into two groups: high growth potential (Risser 0–1, n = 42) and near skeletal maturity (Risser 4–5, n = 92). Both groups demonstrated comparable reductions in Cobb angle at 6 and 12 months. The analysis focused on these two extremes of skeletal maturity to avoid redundancies, as patients with intermediate maturity demonstrated outcomes comparable to those of the adjacent groups and were not presented separately. At 12 months, mean reductions were −3.7° (95% CI −4.1 to −3.3) in the Risser 0–1 group and −3.8° (95% CI −4.0 to −3.6) in the Risser 4–5 group. The adjusted between-group difference was 0.1° (95% CI −0.2 to 0.4; *p* = 0.63), indicating no significant difference in treatment response according to skeletal maturity. Detailed subgroup data are presented in Table 5.

When outcomes were analyzed by curve pattern (thoracic, thoracolumbar, lumbar, and double major), no statistically significant differences were observed in the magnitude of Cobb angle reduction or ATR improvement.

### 3.5. Month-to-Month Consistency

Monthly clinical follow-ups (scoliometer readings, postural grid assessment, and trunk mobility tests) suggested gradual, steady improvement throughout the 12-month program, with no obvious regressions observed between scheduled radiographic assessments. These interim evaluations served to monitor adherence and detect potential setbacks but were not subjected to statistical testing.

Radiographic comparisons confirmed significant reductions at 6 and 12 months, with gains maintained between these two time points (Table 6).

### 3.6. Influence of Age and Sex on Improvement

Cobb angle improvement over 12 months did not differ significantly by age or sex, and no interaction was observed (Table 7).

Similarly, improvements were comparable across Risser stages, indicating that treatment effects were independent of skeletal maturity (Table 8).

### 3.7. Multivariable Regression Analysis

To further examine predictors of treatment response, we performed a multiple linear regression with change in Cobb angle (baseline → 12 months) as the dependent variable and baseline severity, age, sex, and Risser stage as predictors. The model confirmed that baseline severity was the only significant predictor of improvement (β = −0.42, 95% CI (−0.58, −0.26), *p* < 0.001). Neither age (*p* = 0.41), sex (*p* = 0.67), nor Risser stage (*p* = 0.53) were significant predictors (Table 9).

These results reinforce that patients with milder baseline curves experienced greater absolute reductions in Cobb angle, independent of demographic or skeletal maturity factors.

### 3.8. Clinically Meaningful Improvement

A total of 24.3% of patients achieved a Cobb angle reduction of ≥20%, while the remainder showed smaller but still significant improvements (Table 10).

### 3.9. Trunk Rotation

ATR decreased significantly from 7.2° at baseline to 5.6° at 6 months and 5.1° at 12 months (Table 11). Post hoc tests confirmed significant reductions from baseline to both follow-up points, with a smaller but significant change between 6 and 12 months. Overall, pre–post reduction was −2.1° (*p* < 0.001), and improvements in ATR correlated moderately with reductions in Cobb angle (r = 0.42, *p* < 0.001).

## 4. Discussion

This study investigated the effects of a structured 12-month physical therapy program designed to improve spinal alignment in patients with idiopathic scoliosis. The findings demonstrate that conservative, exercise-based therapy can produce meaningful changes, with nearly one in four participants achieving a clinically relevant reduction in Cobb angle. These results reinforce the role of structured physical therapy as an effective management option, particularly for patients with milder curves.

Importantly, nearly one-quarter of participants achieved a clinically meaningful improvement (≥20% reduction), highlighting the practical relevance of the intervention. These results are consistent with a recent comprehensive meta-analysis, which confirmed the corrective potential of structured exercise programs in AIS [23].

The temporal pattern of change, characterized by rapid early gains followed by a plateau, aligns with previous reports of neuromuscular adaptation and postural consolidation during conservative scoliosis management [34,35]. Improvements were consistent month-to-month, with no evidence of regression, underscoring the durability of therapeutic benefits when adherence is maintained [36,37,38].

Subgroup analyses demonstrated that patients with milder baseline curves achieved greater absolute improvements in Cobb angle. This effect persisted in multivariable regression, where baseline severity remained the only significant predictor of outcome, while age, sex, and skeletal maturity were not significant. These results confirm that baseline curve magnitude is the dominant determinant of treatment response, independent of demographic or maturational factors. Clinically, this underscores the importance of early intervention in patients with smaller curves, who appear to derive the greatest benefit from structured exercise therapy [14,34,39].

### 4.1. Interpretation of Spinal Alignment Improvements

Participants demonstrated both statistically and clinically significant reductions in Cobb angle over the 12-month program, with a mean decrease of 3.74° (approximately 20% relative improvement). Although this absolute change did not reach the ≥5° minimum clinically important difference recommended in some guidelines, it exceeded the ≥2° threshold generally regarded as clinically meaningful in conservative scoliosis management and is particularly relevant in older adolescents and young adults with limited remaining growth potential [40,41].

These findings are consistent with previous reports showing that structured, individualized exercise programs can enhance spinal alignment. Comparable reductions in Cobb angle have been reported following physiotherapeutic scoliosis-specific exercises [7,37]. Improvements in posture and quality of life with the addition of structured scoliosis-specific exercises interventions to standard care have also been demonstrated [38,41]. More recent work has highlighted positive effects on trunk rotation and appearance with conservative interventions, although reductions in Cobb angle were less pronounced [36]. In contrast, rigid bracing has been associated with greater curve reduction but often with lower adherence.

It is important to note that the present intervention was not PSSE- or Schroth-based. Rather, it was a structured physiotherapy program incorporating postural correction, Klapp-based positioning, and core stabilization principles. Despite methodological differences, the observed outcomes are comparable in magnitude to those reported for PSSE approaches, suggesting that general physiotherapy principles—when applied systematically and with high adherence—can also achieve clinically meaningful improvements.

Taken together, the evidence indicates that structured physiotherapy can yield clinically meaningful improvements in spinal alignment while avoiding some of the limitations associated with brace treatment. The large effect size observed (Cohen’s dz = 4.15) suggests a robust treatment response, reflecting true physiological adaptation and maintained correction rather than a statistical artifact [34,42,43,44].

### 4.2. Improvement in Trunk Rotation

In addition to radiographic outcomes, significant reductions in trunk rotation were observed during the 12-month intervention, with the steepest improvements occurring in the first six months and closely paralleling the trajectory of Cobb angle reduction. A decrease of more than 2° in ATR is clinically meaningful, as trunk rotation captures the three-dimensional deformity of scoliosis and directly influences cosmetic appearance, rib hump prominence, and patient-reported outcomes [45,46,47].

The moderate correlation between changes in ATR and Cobb angle underscores their complementary value: while the Cobb angle remains the gold standard for assessing coronal curvature, ATR provides critical insight into axial deformity and functional correction. Recent studies have emphasized that incorporating trunk rotation measures alongside radiographic outcomes allows a more holistic assessment of conservative scoliosis treatment [20,48,49].

By integrating both radiographic and clinical measures, this study contributes to a more comprehensive understanding of the effects of structured physical therapy on scoliosis, supporting calls from recent literature to evaluate three-dimensional correction rather than relying solely on coronal plane measurements [50,51].

### 4.3. Analysis of the Temporal Pattern of Improvement

The greatest improvements in Cobb angle occurred during the first six months of therapy, with subsequent changes being more gradual. This temporal trajectory is consistent with other longitudinal rehabilitation studies, where early gains have been attributed to intensive motor relearning, enhanced proprioceptive control, and strong patient adherence during the initial phase of structured exercise programs [34,41,52,53].

The plateau observed after approximately Month 6 aligns with evidence suggesting that once postural corrections are established, further changes tend to progress more slowly as the therapeutic emphasis shifts from structural realignment toward functional adaptation and consolidation [54,55,56]. By the final months of therapy, improvements appeared to stabilize, reflecting a maintenance phase rather than continued correction.

Clinically, these findings highlight the first half-year of therapy as a critical window for maximizing spinal realignment, while the latter phase serves primarily to consolidate progress and prevent regression, a pattern also described in other conservative scoliosis interventions.

### 4.4. Insights into Improvement by Baseline Severity

Contrary to Hypothesis 3, patients with lower baseline Cobb angles (<18.6°) demonstrated greater absolute improvement than those with higher initial deviations, although both groups achieved statistically significant reductions. This observation contrasts with earlier rehabilitation studies suggesting that larger initial curvatures may provide greater scope for measurable correction [9,15,57].

A likely explanation is that milder curves are less rigid and therefore more adaptable to postural adjustments within the same treatment period. In contrast, more advanced curvatures may involve structural remodeling of vertebrae and soft tissues that reduce responsiveness to conservative interventions. Previous studies have similarly noted that longer disease duration, reduced spinal flexibility, and increased biomechanical constraints limit the corrective potential in patients with more severe scoliosis [9,58,59].

Taken together, these findings underscore the importance of early detection and timely initiation of therapy. Commencing structured physical therapy before scoliosis progresses to more advanced stages may maximize the potential for clinically meaningful correction, a conclusion echoed by both exercise- and brace-based studies [60,61,62].

### 4.5. Evaluation of Month-to-Month Consistency

Alignment improvements were maintained consistently across the 12-month program, with no evidence of regression. Radiographic assessments confirmed significant reductions at both 6 and 12 months, and interim clinical evaluations (scoliometer checks, posture grid assessments, and trunk mobility tests) demonstrated steady progress between these time points. These interim assessments served primarily for monitoring, while formal statistical analyses focused on radiographic outcomes.

The largest reduction occurred during the first six months, followed by smaller but still meaningful improvements in the latter half of the program. This trajectory reflects the typical neuromuscular adaptation curve: rapid early gains associated with enhanced muscle activation, proprioceptive recalibration, and postural re-education, followed by a plateau as the spine approaches maximal achievable correction and soft tissue remodeling slows [11,63,64]. These findings are consistent with prior longitudinal studies demonstrating that regular participation in structured exercise programs prevents regression and preserves alignment benefits in patients with scoliosis [64,65,66]. Clinically, this underlines the importance of adherence to therapy, not only to achieve but also to sustain improvements over time.

### 4.6. Influence of Age, Sex, and Skeletal Maturity on Improvement

Cobb angle improvements did not differ significantly by age or sex, and skeletal maturity as assessed by the Risser score was not associated with treatment response. These findings suggest that structured physical therapy provides benefits across demographic and maturational subgroups, supporting its broad applicability in clinical practice.

To address the baseline imbalance between skeletal maturity groups, subgroup analyses were performed. Patients with high growth potential (Risser 0–1) and those near skeletal maturity (Risser 4–5) showed comparable improvements at 6 and 12 months, with no significant between-group differences. These findings indicate that treatment effects were not materially influenced by skeletal maturity, reinforcing the robustness of the overall outcomes.

These observations align with recent studies reporting that scoliosis-specific exercise interventions can be effective regardless of sex or age, provided that protocols are individualized and adherence is maintained [42,67]. While younger patients may theoretically benefit from greater spinal flexibility and adaptability, emerging evidence shows that adults with scoliosis can also achieve clinically relevant improvements, particularly in posture, pain, and quality of life [45,68,69].

Nevertheless, it must be acknowledged that skeletal maturity provides a more precise indicator of progression risk and therapeutic responsiveness than chronological age alone. For instance, a 10-year-old child with Risser 0 differs fundamentally from a 15-year-old adolescent with Risser 4 in terms of growth potential, curve progression risk, and adaptability to exercise. Although our results did not reveal significant differences across Risser stages, this biological distinction remains clinically important and should be considered when interpreting treatment outcomes. Future research should further examine whether the magnitude or nature of exercise responsiveness varies by skeletal maturity, beyond the chronological age categories applied here.

Traditionally, conservative therapy has been regarded as most effective before skeletal maturity, when spinal plasticity is greatest. However, these findings challenge that assumption by demonstrating comparable improvements among older adolescents and adults. This reinforces the view that while early initiation remains preferable, structured physiotherapy should not be excluded after maturation, as it continues to provide measurable benefits for patients across the age spectrum [63,66,69,70].

### 4.7. Considerations on Clinically Meaningful Improvement

Nearly one-quarter of participants (24.3%) achieved a ≥20% reduction in Cobb angle after 12 months of therapy. This threshold is widely recognized as an indicator of substantial postural correction and functional benefit in scoliosis rehabilitation [40,71]. While the majority of patients did not reach this benchmark, even smaller reductions may contribute to meaningful improvements in pain, mobility, and quality of life, as reported in previous clinical trials [72,73,74].

The fact that nearly one in four patients achieved such substantial correction without surgical intervention underscores the potential of structured physical therapy to deliver clinically relevant outcomes within a relatively short timeframe. Comparable proportions of responders have been observed in other recent longitudinal studies of scoliosis-specific exercise, further reinforcing the role of conservative therapy as a viable first-line treatment option [12,75,76].

These findings highlight the value of targeted, sustained physiotherapy not only in slowing curve progression but also in achieving clinically significant correction in a subset of patients. They also emphasize the importance of early identification of individuals most likely to benefit from intensive conservative interventions, an approach increasingly advocated in contemporary rehabilitation guidelines [14,77,78].

Similarly, when treatment outcomes were analyzed by curve pattern (thoracic, thoracolumbar, lumbar, and double major), no statistically significant differences were observed in the magnitude of Cobb angle or ATR improvement. This suggests that the structured physiotherapy program was comparably effective across different curve morphologies, consistent with the view that general corrective exercise principles can benefit a broad range of scoliosis patterns.

### 4.8. Implications for Quality of Life

The structural improvements observed in Cobb angle and trunk rotation may have important implications for patient quality of life. Previous studies have demonstrated that even modest reductions in spinal curvature are associated with improved self-image, reduced rib hump prominence, and better psychosocial outcomes in individuals with scoliosis [76,79,80]. Exercise-based interventions have also been linked to reductions in pain and functional limitations, further contributing to health-related quality of life [40,66,75].

Although this study did not directly assess quality of life using standardized instruments such as the SRS-22r or SF-36, the observed radiographic and postural improvements are consistent with the types of changes that prior research has shown to translate into meaningful functional and psychosocial benefits. These findings support the growing evidence base that conservative physiotherapy programs can improve not only structural outcomes but also patient well-being.

### 4.9. Clinical Implications

These findings carry several implications for conservative scoliosis management. The significant and sustained reductions in Cobb angle confirm that structured physical therapy can serve as an effective alternative or adjunct to bracing, particularly for mild-to-moderate curves. The pronounced improvements during the first six months highlight this period as a critical therapeutic window, underscoring the importance of high-intensity engagement early in treatment.

The absence of differences by age or sex supports the broad applicability of this protocol across diverse patient groups, facilitating clinical adoption without major modifications. Although only about one-quarter of participants achieved a ≥20% reduction in Cobb angle, the smaller improvements observed in others may still translate into functional and quality-of-life gains. This suggests that clinicians should evaluate both angular change and patient-reported outcomes when assessing treatment success.

It should also be acknowledged that for patients aged 15 years and older—particularly girls with mild or moderate curves—the risk of progression is generally lower compared with younger adolescents. Nevertheless, some residual progression can still occur in late adolescence, especially in those who have not reached full skeletal maturity or present with curves near the 25° threshold. Moreover, even in the absence of progression, scoliosis can affect posture, trunk symmetry, and self-image. In this context, our results support the role of structured physical therapy not only in reducing progression risk but also in improving functional and cosmetic outcomes in this older age group.

Finally, the plateau observed after Month 6 indicates the need for periodic reassessment of therapy goals and strategies. Adjusting exercise intensity, reinforcing adherence, and incorporating personalized progression plans and patient education may help sustain motivation and maximize long-term benefits while reducing the risk of curve progression.

Including patients over 18 years in the study reflects the reality of clinical practice, where many individuals first seek conservative treatment in late adolescence or adulthood. Our results demonstrate that structured physical therapy remains relevant in this population, providing not only stabilization but also measurable improvements in posture, trunk symmetry, and quality of life.

### 4.10. Study Limitations and Future Research

While this study provides strong evidence for the effectiveness of a structured physical therapy program in scoliosis management, several limitations must be acknowledged. First, the absence of a control group limits causal inference. Although the consistent within-patient improvements observed suggest that spontaneous recovery alone is unlikely, randomized controlled trials remain necessary to confirm causality and rule out residual confounding.

Second, follow-up was restricted to 12 months, leaving uncertainty about whether benefits are durable in the longer term. Extended follow-up studies are warranted to determine the sustainability of improvements and the potential role of maintenance programs.

Third, the intervention represented a structured physiotherapy program reflecting current clinical practice, rather than a replication of any single standardized or proprietary method. It integrated elements of postural correction, Klapp-based positioning, and core stabilization, together with corrective breathing and three-dimensional alignment strategies. While some of these components conceptually overlap with PSSE principles, the present program was not PSSE- or Schroth-certified. This pragmatic design mirrors real-world physiotherapy practice but may limit direct comparability with studies that strictly follow school-specific exercise protocols.

Fourth, the sample was drawn from a limited number of specialized treatment centers, which may restrict generalizability. Although attrition was low (7.5%), differences between completers and non-completers cannot be excluded. Moreover, the high adherence rate (>90%) may reflect favorable clinical conditions (e.g., strong therapist-patient rapport, convenient access) and could overestimate adherence in less controlled or community-based settings.

Fifth, while the ATR provided valuable insight into three-dimensional correction, scoliometer-based measurement is examiner-dependent and reflects surface asymmetry rather than true vertebral rotation, introducing potential variability. In addition, outcomes focused primarily on radiographic and postural measures; the absence of functional endpoints such as pain, mobility, respiratory function, and quality of life (as discussed above) limits the scope of conclusions. Including these outcomes in future studies would provide a more comprehensive evaluation of therapeutic benefit.

Another limitation of our study is the restriction of inclusion to patients aged ≥ 15 years. While this criterion was chosen to minimize variability related to skeletal immaturity and to ensure consistency in adherence assessment, it may limit the generalizability of findings to younger adolescents (10–14 years) who also represent an important target group for conservative scoliosis management. Future studies should therefore include younger patients to examine whether the observed effects are comparable across developmental stages.

In addition, although the mean Cobb angle reduction (3.7°) was statistically significant, it did not reach the ≥5° threshold that some guidelines define as a minimum clinically important difference. This should be interpreted with caution. However, the relative reduction (20.3%) exceeded the 20% benchmark frequently used in scoliosis rehabilitation research, and nearly one-quarter of patients achieved this level of clinically meaningful improvement. Future research should further examine how absolute versus relative thresholds apply across different age groups and levels of skeletal maturity.

Finally, the use of chronological age categories (<18 vs. ≥18 years) for subgroup analyses is another limitation, as they imperfectly reflect skeletal maturity. Skeletal maturity assessed by the Risser score is a more precise determinant of progression risk and responsiveness to therapy. In this study, inclusion of Risser staging partly addressed this issue, revealing consistent improvements across maturity levels.

Future research should prioritize randomized or controlled designs, extend follow-up over one year, and investigate the optimal exercise dosage and progression strategies. Evaluating cost-effectiveness and integrating both objective outcomes (radiographic and three-dimensional assessments) and patient-reported measures will allow for a more holistic appraisal of conservative scoliosis interventions. Such efforts are essential to strengthen the evidence base and facilitate the translation of structured physical therapy into routine clinical practice.

## 5. Conclusions

This prospective 12-month observational study demonstrates that a structured physical therapy program can produce statistically and clinically significant improvements in spinal alignment among patients with scoliosis. On average, the Cobb angle decreased by 3.7°, with the greatest gains observed during the first six months and smaller but sustained improvements thereafter. Month-to-month evaluations confirmed steady progress with minimal regression.

Contrary to initial expectations, patients with milder baseline curves achieved greater absolute improvements than those with more severe deviations, while age, sex, and skeletal maturity were not significant predictors of outcome. Nearly one-quarter of participants achieved a clinically meaningful reduction of at least 20%, underscoring the practical relevance of this intervention. Improvements in trunk rotation further confirmed its three-dimensional corrective effect.

Although the average absolute change (3.7°) did not reach the ≥5° minimum clinically important difference recommended in some guidelines, it exceeded the 2° threshold commonly regarded as relevant in non-surgical management. Moreover, the relative reduction of 20.3% and the proportion of patients surpassing the ≥20% benchmark highlight that the observed changes are clinically meaningful, particularly in a cohort of older adolescents and young adults with limited remaining growth potential.

These findings support the role of targeted, exercise-based therapy as an effective, non-invasive, and broadly applicable option for scoliosis management in real-world clinical practice. Importantly, the absence of differences by age or skeletal maturity suggests that physiotherapy remains valuable even after 18 years of age, challenging the traditional assumption that therapeutic interventions lose relevance once skeletal maturation is complete.

Although the absence of a control group limits causal inference, the consistent within-patient improvements observed across multiple subgroups provide compelling evidence of therapeutic benefit. Early and sustained engagement appears critical for maximizing outcomes, while ongoing follow-up may help preserve alignment gains. Future studies should evaluate the long-term durability of improvements, refine exercise protocols, and incorporate functional and patient-reported outcomes to complement radiographic assessments.

## Figures and Tables

**Table 1:** Baseline characteristics of the study participants (n = 222 completers).

Characteristic	n (%) or Mean ± SD
Age (years)	16.8 ± 1.2 (range: 15–19)
<18 years	112 (50.5)
≥18 years	110 (49.5)
Sex	
Male	108 (48.6)
Female	114 (51.4)
Cobb angle (°)	18.6 ± 0.4 (15.0–25.0)
ATR (°)	7.2 ± 1.1 (5.0–9.4)
Risser stage	
0–1	42 (18.9)
2–3	88 (39.6)
4–5	92 (41.4)
Curve pattern	
Thoracic	102 (45.9)
Thoracolumbar	69 (31.1)
Lumbar	38 (17.1)
Double major	13 (5.9)
Initially enrolled	240 (100.0)
Completed 12 months	222 (92.5)

Note: Age, Cobb angle, and ATR values are presented as mean ± SD with min–max; percentages for age, sex, Risser stage and curve pattern are based on completers.

**Table 2:** Pre–post change in spinal alignment (Cobb angle).

Time Point	N	Mean ± SD (Min–Max)	Δ (95% CI)	t (df)	*p*	dz
Baseline	222	18.59 ± 0.40 (15.0–25.0)	—	—	—	—
6 months	222	15.55 ± 0.92 (13.5–18.2)	−3.04 (−3.15 to −2.93)	58.47 (221)	<0.001	3.90
12 months	222	14.85 ± 1.29 (12.3–18.5)	−3.74 (−3.86 to −3.63)	61.86 (221)	<0.001	4.15

Note: Δ = change score (Month 12 − Baseline); dz = Cohen’s d for paired samples.

**Table 3:** Pairwise comparisons of Cobb angle change (Bonferroni-adjusted).

Comparison	Mean Difference (°)	*p*-Value (Bonferroni)
Baseline → 6 months	−3.04	<0.001
Baseline → 12 months	−3.74	<0.001
6 months → 12 months	−0.70	0.012

Note: Negative values indicate a reduction in Cobb angle.

**Table 4:** Cobb angle improvement by baseline severity.

Severity Group	N	Mean Improvement ± SD (Min–Max)
High baseline deviation (≥18.6°)	127	3.21 ± 0.67 (1.9–4.6)
Low baseline deviation (<18.6°)	95	4.45 ± 0.65 (3.2–5.8)

Note: Improvement calculated as (Baseline − Month 12). Positive values indicate a reduction in Cobb angle.

**Table 5:** Changes in Cobb Angle Over 12 Months by Skeletal Maturity Group.

Time Point	Risser 0–1 (n = 42)	Risser 4–5 (n = 92)	Adjusted Mean Difference (95% CI)	*p*-Value
Baseline (°)	18.6 ± 0.5	18.5 ± 0.4	—	—
6 months Δ (°)	−3.1 (95% CI −3.5 to −2.7)	−3.0 (95% CI −3.2 to −2.8)	0.1 (−0.2 to 0.4)	0.54
12 months Δ (°)	−3.7 (95% CI −4.1 to −3.3)	−3.8 (95% CI −4.0 to −3.6)	0.1 (−0.2 to 0.4)	0.63

Note: Δ indicates the change from baseline. Values represent mean change from baseline with 95% confidence intervals. Between-group differences were estimated using linear models adjusted for baseline Cobb angle, age, and sex. The intermediate Risser 2–3 group showed comparable results and was not presented separately for clarity.

**Table 6:** Radiographic pairwise changes in Cobb angle.

Comparison	Mean Change (°)	*p*-Value
Baseline → 6 months	−3.04	<0.001
Baseline → 12 months	−3.74	<0.001
6 months → 12 months	−0.70	0.012

Note: Negative values indicate a reduction in Cobb angle. Clinical evaluations confirmed steady progress with no evidence of regression between radiographic assessments.

**Table 7:** Two-way ANOVA for improvement in Cobb angle by age group and sex.

Source	df	SS	MS	F	*p*
Age group	1	0.895	0.895	1.09	0.297
Sex	1	0.019	0.019	0.02	0.878
Age × Sex	1	0.279	0.279	0.34	0.560

Note: SS = sum of squares; MS = mean square.

**Table 8:** Cobb angle improvement by Risser stage.

Risser Stage	N	Mean Improvement ± SD (Min–Max)
0–1	42	3.8 ± 0.9 (2.0–5.6)
2–3	88	3.6 ± 1.0 (1.6–5.6)
4–5	92	3.7 ± 1.1 (1.5–5.9)

Note: Improvement = Baseline − Month 12. Positive values indicate a reduction in Cobb angle.

**Table 9:** Multivariable regression predicting change in Cobb angle.

Predictor	β (Coefficient)	95% CI	*p*-Value
Baseline severity	−0.42	−0.58 to −0.26	<0.001
Age	0.05	−0.07 to 0.16	0.41
Sex (Male vs. F)	0.03	−0.11 to 0.17	0.67
Risser stage	−0.04	−0.18 to 0.09	0.53

Note: Dependent variable = change in Cobb angle (Baseline − 12 months). Negative values indicate greater improvement in patients with milder baseline curves.

**Table 10:** Patients achieving ≥ 20% Cobb angle reduction.

Outcome Category	N	%
≥20% reduction from baseline	54	24.3
<20% reduction from baseline	168	75.7

**Table 11:** ATR over the 12-month program.

Time Point	N	Mean ATR (°)
Baseline	222	7.2 ± 1.1 (5.0–9.4)
6 months	222	5.6 ± 1.0 (3.6–7.6)
12 months	222	5.1 ± 1.2 (2.7–7.5)

Note: When thoracic and lumbar prominences co-existed, the maximal ATR is reported.

## Data Availability

Data supporting the findings of this study can be obtained from the corresponding author upon reasonable request. Public access to the datasets is restricted to protect the privacy of adolescent participants and to comply with ethical regulations.

## Abbreviations

The following abbreviations are used in this manuscript:

PSSE	Physiotherapeutic scoliosis-specific exercises
ICC	Intraclass correlation coefficient
ATR	Angle of trunk rotation
AIS	Adolescent idiopathic scoliosis
SEAS	Scientific exercise approach to scoliosis
